# Tumor Membrane Vesicle Vaccine Augments the Efficacy of Anti-PD1 Antibody in Immune Checkpoint Inhibitor-Resistant Squamous Cell Carcinoma Models of Head and Neck Cancer

**DOI:** 10.3390/vaccines8020182

**Published:** 2020-04-14

**Authors:** Ramireddy Bommireddy, Luis E. Munoz, Anita Kumari, Lei Huang, Yijian Fan, Lenore Monterroza, Christopher D. Pack, Sampath Ramachandiran, Shaker J.C. Reddy, Janet Kim, Zhuo G. Chen, Nabil F. Saba, Dong M. Shin, Periasamy Selvaraj

**Affiliations:** 1 Department of Pathology and Laboratory Medicine, Emory University School of Medicine, Atlanta, GA 30322, USA; rbommi2@emory.edu (R.B.); luis.munoz@emory.edu (L.E.M.); anitakamal09@gmail.com (A.K.); lei.huang@emory.edu (L.H.); yijian.fan@emory.edu (Y.F.); lenore.monterroza@emory.edu (L.M.); janetkim96@gmail.com (J.K.); 2 Metaclipse Therapeutics Corporation, Atlanta, GA 30340, USA; cpack@metaclipse.com (C.D.P.); sramachandiran@metaclipse.com (S.R.); sreddy@metaclipse.com (S.J.C.R.); 3 Department of Hematology and Medical Oncology, Emory University School of Medicine, Atlanta, GA 30322, USA; gzchen@emory.edu (Z.G.C.); nfsaba@emory.edu (N.F.S.)

**Keywords:** cancer, vaccine, adjuvants, IL-12, TMVs, MOC1, MOC2, SCCVII, HNSCC, anti-PD1

## Abstract

Immune checkpoint inhibitor (ICI) immunotherapy improved the survival of head and neck squamous cell carcinoma (HNSCC) patients. However, more than 80% of the patients are still resistant to this therapy. To test whether the efficacy of ICI therapy can be improved by vaccine-induced immunity, we investigated the efficacy of a tumor membrane-based vaccine immunotherapy in murine models of HNSCC. The tumors, grown subcutaneously, are used to prepare tumor membrane vesicles (TMVs). TMVs are then incorporated with glycolipid-anchored immunostimulatory molecules GPI-B7-1 and GPI-IL-12 by protein transfer to generate the TMV vaccine. This TMV vaccine inhibited tumor growth and improved the survival of mice challenged with SCCVII tumor cells. The tumor-free mice survived for several months, remained tumor-free, and were protected following a secondary tumor cell challenge, suggesting that the TMV vaccine induced an anti-tumor immune memory response. However, no synergy with anti-PD1 mAb was observed in this model. In contrast, the TMV vaccine was effective in inhibiting MOC1 and MOC2 murine oral cancer models and synergized with anti-PD1 mAb in extending the survival of tumor-bearing mice. These observations suggest that tumor tissue based TMV vaccines can be harnessed to develop an effective personalized immunotherapy for HNSCC that can enhance the efficacy of immune checkpoint inhibitors.

## 1. Introduction

Treatment of head and neck squamous cell carcinoma (HNSCC) with immune checkpoint inhibitors (ICIs) has resulted in improved survival of patients with metastatic HNSCC; however, the majority of patients (>80%) fail this treatment regardless of tumor human papilloma virus (HPV) status or demographic factors [1,2,3,4,5,6]. Since ICIs act by releasing the breaks on pre-existing anti-tumor T cell immunity, the lower response rate of HNSCC patients to anti-programmed cell death protein 1 (PD-1 aka CD279) antibody suggests that a large number of HNSCC patients either have low pre-existing anti-tumor immunity or the tumors use immunosuppressive pathways that can inhibit T cell activity. A recent study suggests that HNSCC patients with high T cell infiltrates show improved survival with ICI treatment [7]. Therefore, an effective vaccination approach that can convert tumors with no or few T cell infiltrates (cold tumor) to tumors with high number of T cell infiltrates (hot tumor) would be highly desirable to increase the efficacy of ICIs in HNSCC patients.

Although therapeutic cancer vaccines showed promising results in animal models, they have conferred only modest anti-tumor activity in several human clinical trials for many cancers, including HNSCC [8,9,10]. Many of the previous approaches employed genetically modified allogeneic tumor cell lines or cell lines developed from patients or single tumor antigens or tumor antigen–specific peptides as vaccine sources [11]. These approaches induced anti-tumor immunity in patients, but clinical benefit was not achieved because the tumors might have escaped immune attack due to their heterogeneity, since the vaccines might not have represented all the variations found in the patient’s tumor. It has been shown that the HNSCC tumor from each patient is distinct in its gene mutations and target antigens [12,13,14,15,16,17,18], a major challenge in developing targeted vaccine immunotherapy approaches for these patients. To address these limitations, personalized vaccine immunotherapy approaches that include patient-specific antigens are being developed. These include the use of patient-specific neoepitope peptide vaccines [19,20,21] and whole tumor lysate pulsed dendritic cell (DC) approaches [22,23]. This suggests that it is critical to use patient-specific antigens in developing efficacious therapeutic cancer vaccines. Therefore, our goal is to induce anti-tumor immunity in HNSCC patients using the patient’s own tumor tissue as the multi-antigen source. In this study, we are investigating a tumor membrane vesicle (TMV)-based vaccine immunotherapy approach for HNSCC in syngeneic mouse models. These TMVs are uniform in size (300–500 nm) and are suitable for uptake and processing by antigen presenting cells such as DCs.

Our personalized TMV vaccine immunotherapy uses a novel technology to incorporate glycosyl phosphatidylinositol (GPI)-anchored forms of immunostimulatory molecules (GPI-ISMs) onto TMVs derived from the surgically excised tumor tissue as a vaccine source. Studies by others and our lab have shown that purified GPI-anchored proteins can be spontaneously incorporated onto cell membranes after a short incubation time [24,25]. This technique of incorporating new proteins onto live cell surfaces or isolated membrane vesicles using purified GPI-anchored proteins is referred to here as “protein transfer.” This property of GPI-anchored molecules permits the manipulation of TMVs to display desired ISMs without the use of gene transfer. TMVs expressing such modified GPI-ISMs are capable of inducing protective anti-tumor immunity in breast cancer and thymoma models [26,27]. Since the TMVs contain the unique antigenic profile for each patient tumor, this personalized approach can be an effective immunotherapy for HNSCC, wherein the target antigens vary from patient-to-patient [12,13,14,15,16,17,18].

Our TMV vaccine simultaneously delivers the patient’s unique tumor antigen signature along with potent ISMs to immune cells resulting in effective anti-tumor immunity. We hypothesize that TMV vaccine induces de novo anti-tumor immunity, which enhances the efficacy of ICI immunotherapy to confer protection against recurrence and relapse of metastatic disease. In the present study, we demonstrate that therapeutic TMV vaccine administration inhibits HNSCC tumor growth, prolongs survival, and induces durable anti-tumor immunity in syngeneic murine models. We also show that TMV vaccine in combination with anti-PD-1 antibody is more effective than either single agent against ICI-resistant tumors in HNSCC murine model.

## 2. Materials and Methods

### 2.1. Antibodies

Mouse anti-mouse PD-1 monoclonal antibody was a kind gift from Dr. Gordon Freeman (Dana-Farber Cancer Institute, Harvard University, Boston, MA, USA). Rat anti-mouse PD-1 monoclonal antibody (Catalog# BP0146; Clone RMP1-14) was purchased from BioXCell Inc. (West Lebanon, NH, USA). Purified and Fluorochrome-conjugated antibodies (TruStain FcX anti-mouse CD16/32, Catalog#101319; anti-mouse CD80-FITC, catalog#104705; anti-mouse CD44-FITC, catalog#103005; anti-mouse CD24-PE, catalog#138503; anti-mouseCD47-PE, catalog# 127507; anti-mouse IL-12-PE, catalog#505203; anti-mouse CD8-APC, catalog#100712; anti-mouse Gr1-APC, catalog#108411; anti-mouse PD-L1-APC, catalog#124311; and anti-mouse CD4-BV711, catalog#100447) for flow cytometry analysis were purchased from BioLegend Inc. (San Diego, CA, USA). FITC-conjugated secondary antibodies (goat anti-mouse IgG-FITC, catalog# 115-095062) were purchased from Jackson Immunoresearch Labs (Bar Harbor, ME, USA).

### 2.2. Mice

C3H/HeJ and C57BL/6 mice (Female, 2–3 months old) purchased from Jackson Laboratories (Bar Harbor, ME, USA) were housed in Woodruff Memorial Research Building Division of Animal Resources facility (WMB-DAR) and used in all the experiments. Four-8 mice were included for each treatment group as described in the figure legends. Experiments were conducted according to Emory University Institutional Animal Care and Use Committee (IACUC) approved protocols (DAR-2017-00-504).

### 2.3. Cell Lines

The squamous cell carcinoma VII (SCC VII) tumor cell line [28] was a kind gift from Dr. Jennifer R. Grandis (University of Pittsburgh, Pittsburgh, PA, USA; now at University of California, San Francisco, CA, USA). SCC VII is a moderately well differentiated tumorigenic cell line derived from a spontaneous squamous cell carcinoma of the abdominal cavity in the C3H mouse [29,30]. Murine oral carcinoma-1 and Murine oral carcinoma-2 (MOC1 and MOC2) cell lines were kind gifts from Dr. Ravindra Uppaluri (Dana-Farber Cancer Institute, Harvard University, Boston, MA, USA). MOC1 and MOC2 cell lines are derived from 7,12-dimethylbenz(a)anthracene (DMBA) carcinogen induced oral cancer in C57BL/6 mice [31,32,33]. Tumor cells were cultured in Dulbecco’s Modified Eagle Medium (DMEM) medium with 10% fetal bovine serum.

### 2.4. In Vitro IFN-γ Treatment of Murine HNSCC Cells

MOC1 and MOC2 tumor cells (5 × 10^5^ cells in 3 mL medium) were cultured in six-well tissue culture plates at 37 °C in a CO_2_ incubator with or without 100 ng/mL IFN-γ for 72 hrs. Cells were harvested and surface stained for expression of various surface markers using fluorochrome-conjugated antibodies and analyzed on a FACSCalibur (BD Biosciences, San Jose, CA, USA).

### 2.5. TMV Vaccine Preparation

To obtain tumor tissue for preparing TMV vaccine, MOC1 and MOC2 cells were injected subcutaneously (s.c.) into C57BL/6, and SCC VII cells into C3H/HeJ syngeneic mice. Mice were euthanized, and tumor tissue excised when the tumor size reached IACUC endpoint. Tumor tissue was homogenized, and membranes were isolated by centrifugation over a 41% sucrose gradient. These TMVs were incorporated with immunoaffinity-purified murine GPI-B7-1 and GPI-IL-12 molecules (GPI-ISMs) by protein transfer [26]. GPI-ISM incorporation was confirmed by flow cytometry analysis using fluorochrome-conjugated antibodies.

### 2.6. TMV Vaccine Immunotherapy

C57BL/6 and C3H/HeJ mice were administered with TMV vaccine in sterile PBS subcutaneously three days after challenging the mice with MOC1, MOC2, or SCC VII tumor cells as described in the figure legends. TMV vaccine dose and schedule are described in the figures and figure legends.

### 2.7. Cellular Phenotyping of Immune Infiltrates

SCC VII tumor cells were harvested and resuspended at 2 × 10^5^ cells/mL in serum-free medium. 250 μL of tumor cells (5 × 10^4^) were mixed at a 1:1 ratio with 250 μL of Matrigel (Corning Inc. Tewksbury, MA, USA). The Matrigel-tumor cell mixture (500 μL) was injected into the right hind flank of C3H/HeJ mice [34]. Three days after tumor cell inoculation, mice were administered s.c. with 100 μg of TMV vaccine or 100 μL PBS (control group) every week for four doses. Five days after the last dose of vaccine, Matrigel was harvested and disintegrated by scalpels and forceps. Single cell suspensions were then prepared from Matrigel. Cells were washed three times with media and resuspended in FACS buffer for cell surface staining.

### 2.8. Flow Cytometry

Cell surface markers were analyzed by flow cytometry. Briefly, cells isolated from in vivo studies were pre-incubated with Fc receptor blocking antibody in FACS buffer (PBS with 2% BCS, 5 mM EDTA, 0.05% sodium azide) at 4 °C for 10 min to block nonspecific binding of monoclonal antibodies to immune cells. Fluorochrome-conjugated primary antibodies were added and incubated for 30 min with shaking at 4 °C. Cells were washed three times with FACS buffer and analyzed the cells using a FACSCalibur, BD LSRII (BD Biosciences, San Jose, CA, USA) or a CYTEK Aurora (CYTEK, Fremont, CA, USA) flow cytometer [26,34]. Protein expression profiles of TMVs were also analyzed by staining with fluorochrome-conjugated antibodies after blocking the Fc receptors with Fc blocking antibody [26]. Data were analyzed using FlowJo software ((Becton, Dickinson and Co., Ashland, OR, USA).

### 2.9. Statistical Analysis

Student’s t-test was used for determining the significance between control and treated groups tumor size. Log-rank (Mantel-Cox) test was used for determining the significance of the difference in the survival of mice.

## 3. Results

### 3.1. GPI-B7-1 and GPI-IL-12 Are Incorporated into TMV by Protein Transfer

**Figure 1:**
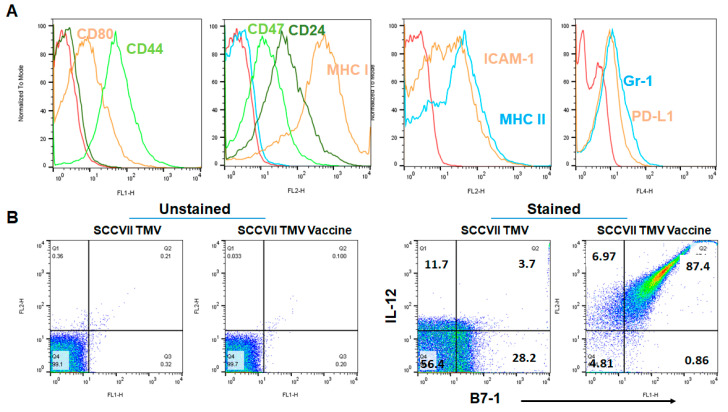
GPI-B7-1 and GPI-IL-12 are incorporated into tumor membrane vesicle (TMV) by protein transfer. Squamous cell carcinoma VII (SCC VII) tumors grown in C3H/HeJ mice were used to prepare TMV. TMVs were incorporated with GPI-B7-1 and GPI-IL-12 by incubating TMVs with purified mouse GPI-B7-1 and mouse GPI-IL-12 at 37 °C for protein transfer as described in the Methods section. Flow cytometry analysis of markers on TMVs (**A**), and protein transferred GPI-B7-1 and GPI-IL-12 on TMV vaccine (**B**) stained with fluorochrome-conjugated antibodies after Fc receptor blocking. Isotype antibodies (**A**, dark green in FL-1 panel, blue in FL-2 panel containing CD24, CD47 and MHC class I) and unstained TMV and TMV vaccine (**B**), left panels were used for gating purposes. A similar procedure was used for preparing MOC1 and MOC2 tumor TMV vaccines. Red histograms in **A** represent unstained TMV.

To test whether TMV vaccine inhibits squamous cell carcinoma growth, we produced a syngeneic cancer vaccine from frozen tumor tissues and isolated TMVs. TMVs are microparticles, which can be visualized by flow cytometry for the expression of various membrane-bound proteins using fluorochrome-conjugated antibodies. Flow cytometry analysis of protein expression profile of unmodified TMVs showed that TMVs express tumor and immune cell markers such as cancer stem cell markers (CD24 and CD44), co-stimulation (CD80 (B7-1)), immunosuppression (CD47, PD-L1), and innate immune cells (Gr1) (Figure 1A). However, the T cell marker CD3 and B cell marker CD19 were not detected on the TMVs. The TMVs were incorporated with purified GPI-B7-1 and GPI-IL-12 by protein transfer to produce TMV vaccine. Flow cytometry analysis of the TMV vaccine confirmed the protein transfer-mediated incorporation of GPI-B7-1 and GPI-IL-12 into TMVs (Figure 1B right panels).

### 3.2. Efficacy of Anti-PD1 Antibody Therapy in SCC VII Tumor Model Depends on the Timing of the Administration

Recent studies suggest that SCC VII tumor cells do not respond to anti-PD-L1 therapy alone but synergize with a mitogen-activated protein kinase kinase (MEK) inhibitor [35]. To determine whether blocking PD-1 with anti-PD-1 antibody inhibits SCC VII tumor growth, we challenged mice with SCC VII cells s.c. and then injected them with anti-PD-1 antibody (Clone RMP1-14;200 μg/dose) intraperitoneally (i.p.) starting eight days after tumor challenge. Three doses of anti-PD-1 antibody therapy completely inhibited SCC VII tumor growth (Supplementary Figure S1A). To confirm whether anti-PD-1 antibody treatment induced protective anti-tumor immunity, tumor-free mice were rechallenged with SCC VII cells 4 months after the primary challenge. These mice were confirmed to be tumor-free, suggesting a protective response. However, anti-PD-1 antibody therapy failed to inhibit SCC VII tumors when the antibody therapy was started 12 days or three weeks after tumor challenge.

### 3.3. TMV Vaccine Inhibits Syngeneic SCC VII Squamous Cell Carcinoma Tumor Growth

In order to determine whether TMV vaccine protects mice from tumor challenge in a prophylactic setting, C3H/HeJ mice were administered with TMV vaccine every week for 4 doses and then challenged with SCC VII tumor cells s.c. one week after the last dose of vaccine administration (Supplementary Figure S2A). We have observed that SCC VII tumor growth was delayed in mice vaccinated with TMV vaccine compared to PBS treated control mice (Supplementary Figure S2B,C). In order to determine whether TMV vaccine induces anti-tumor antibody response, plasma was isolated and analyzed for antibody using SCCVII cells as target and FITC-conjugated anti-mouse IgG as secondary antibody by flow cytometry. However, we did not observe any antibody in the plasma collected after two doses of TMV vaccine. To test whether TMV vaccine inhibits tumor growth in a therapeutic setting, SCC VII tumor-bearing mice were administered with TMV vaccine (100 µg/dose) s.c. on the right flank every week starting three days after tumor cell challenge on the left flank (d3, d10 for two doses or d3, d10, d17 and d24 for four doses). All five mice in the PBS control group developed tumors and reached the IACUC endpoint by day 40. While two doses of TMV vaccine failed to inhibit the tumor growth, four doses of TMV vaccine inhibited tumors in 2/5 mice, delayed tumor growth in 1/5 mouse, and had no effect in 2/5 mice. Tumor-free mice that survived for more than 60 days were rechallenged with SCC VII tumor cells, and tumor growth was monitored.

While all the control naïve mice developed tumors, none of the protected mice in the anti-PD-1 mAb group developed secondary tumor growth (Supplementary Figure S1), suggesting a long lasting protective anti-tumor memory response induced by the immunotherapy.

### 3.4. Multiple Doses of TMV Vaccine Are More Effective than a Single Dose of Bulk TMV Vaccine in Inhibiting SCC VII Tumor Growth

**Figure 2:**
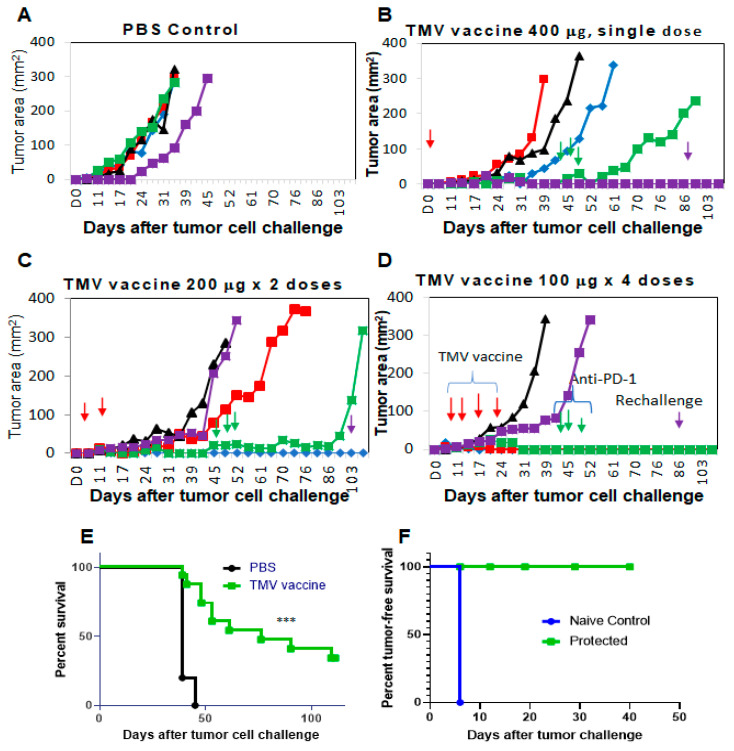
Multiple doses of TMV vaccine is more effective than single dose of bulk TMV vaccine in inhibiting SCC VII tumor growth: C3H/HeJ mice (*n* = 5/group) were injected with 5 × 10^4^ SCC VII cells s.c. and TMV vaccine (red arrows, 100, 200, or 400 μg) injected after three days. (**B**–**D**) Total amount of TMV vaccine per mouse is 400 μg (one time on d3 (**B**), two times on d3 and d10 (**C**) or every week for four doses (d3, d10, d17, and d24 (**D**)). While the tumors have reached IACUC end point in all the mice in control group by d45 (**A**), only four mice from the TMV vaccine groups (**B**–**D**) were euthanized due to the tumor size reaching end point. All remaining mice in the vaccine groups (11/15) were administered with 200 μg anti-PD-1 antibody/mouse/dose (clone RMP1-14) starting on d45 (green arrows, three doses in one week). (**E**) Survival of mice from all the groups that received TMV vaccine was monitored, and (**F**) tumor-free mice (*n* = 5) were rechallenged with SCC VII cells on day103 after first challenge (purple arrows). (**A**–**D**) Each line represents a mouse in the group, different colors and shapes are used for clarity. Log-rank (Mantel–Cox) test was used for determining the significance of the difference in the survival of mice. *** *p* < 0.0001.

In order to determine the effect of dose and dosing schedule on the efficacy of TMV vaccine, we have administered C3H/HeJ mice with TMV vaccine s.c. three days after SCC VII cells were implanted s.c. Total amount of TMV vaccine administered per mouse is 400 μg as 400 μg once on d3 or 200 μg × 2 times on d3 and d10 or 100 μg every week for four doses on d3, d10, d17, and d24) (Figure 2). While the tumor size reached the IACUC end point in all mice in the control group by day 45 (Figure 2A), only in four out of 15 mice from the TMV vaccine groups reached end point. All the remaining mice (11/15) were administered with 200 μg anti-PD-1 antibody/mouse/dose (clone RMP1-14) starting on day 45 (three doses in one week). Interestingly, three out of five mice that received 4 × 100 μg TMV vaccine dose (Figure 2D) survived tumor-free, whereas only one survived in each group that had received 2 × 200 μg (Figure 2C) or 1 × 400 μg (Figure 2B) dose level, and anti-PD1 antibody did not show any effect on the mice with established tumors (Figure 2C,D). Our data also suggest a significant prolongation of survival of the mice receiving TMV vaccine compared to control group (Figure 2E). The tumor-free mice were protected from secondary challenge with SCC VII cells (Figure 2F) suggesting a long-lasting anti-tumor immune memory response induction by TMV vaccine in these mice.

### 3.5. TMV Vaccine Induces T Cell Infiltration into SCC VII Squamous Cell Carcinoma Tumors

**Figure 3:**
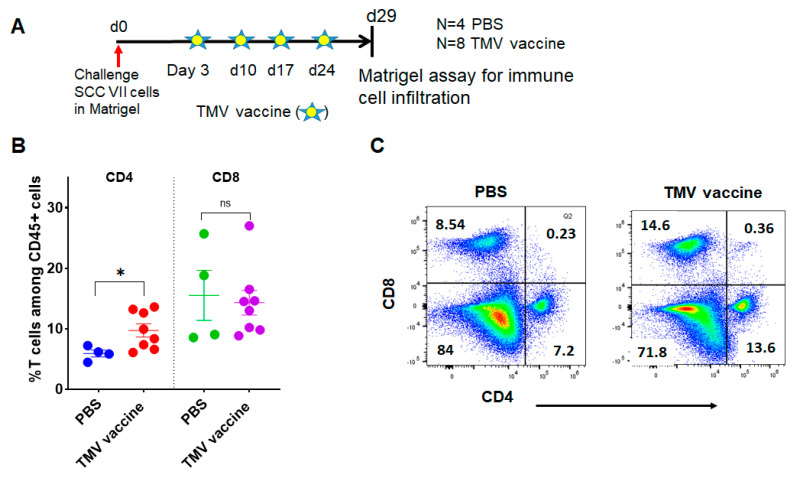
TMV vaccine increases T cell infiltration into SCC VII tumors. SCC VII tumor cells were mixed with Matrigel and the mixture was injected s.c. into the flank of C3H/HeJ mice. TMV vaccine administered every week starting on day 3 after tumor cell inoculation, and the Matrigel was extracted five days after the last dose of TMV vaccine as shown in (**A**). Matrigel plugs were minced with scissors, mashed with plungers, passed through a cell strainer, and extracted cells were processed for flow cytometry to analyze for immune cells using fluorochrome-conjugated antibodies. (**B**) Percentage of CD4^+^ and CD8^+^ T cells among CD45^+^ gated cells, and (**C**) representative flow cytometry analysis plot of tumor infiltrating cells in PBS control and TMV vaccine groups. * *p* < 0.03; ns: not significant.

To test whether TMV vaccine inhibits tumor growth by increasing T cell infiltration, we inoculated mice with SCC VII tumor cells in Matrigel (Corning Inc., Tewksbury, MA, USA) s.c. and then administered TMV vaccine s.c. as shown in Figure 3A. The data show that TMV vaccine significantly increases the percentage of CD4^+^ T cell within the tumor, and, interestingly, no difference was observed in the percentage of CD8^+^ T cells (Figure 3B,C).

### 3.6. TMV Vaccine Enhances Immune Checkpoint Inhibitor Efficacy against MOC Tumors

**Figure 4:**
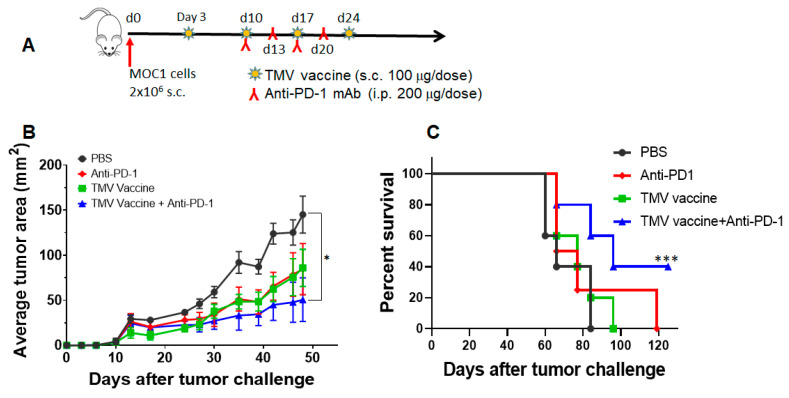
TMV vaccine + anti-PD-1 mAb combination inhibits MOC1 tumor growth. (**A**) C57BL/6 mice (*n* = 5) were challenged with 2 × 10^6^ MOC1 cells (day 0) s.c. on the right flank and they were vaccinated with four doses of 100 µg of TMV vaccine weekly s.c. on the left flank starting on day 3. Rat anti-mouse PD-1 mAb (200 μg/dose) was administered i.p. on d10, d13, d17, and d20 (inverted red Y). (**B**) Tumor size and (**C**) survival are shown. Values are mean ± SD and Student’s *t*-test between PBS and combination group. Log-rank (Mantel–Cox) test was used for determining the significance of the difference in the survival of mice. * *p* < 0.05, *** *p* < 0.001.

To determine whether TMV vaccine inhibits oral SCC tumors in mice, we assessed efficacy of the TMV vaccine on carcinogen-induced murine oral cancer (MOC) cell lines MOC1 and MOC2. MOC1 is a murine oral cancer cell line derived from a DMBA carcinogen induced tumor from C57BL/6 mouse. MOC1 is an indolent tumor with infiltration of CD4^+^ and CD8^+^ T cells and is partially responsive to anti-PD-L1 mAb [31,32,36,37]. MOC2 is also a murine oral cancer cell line derived from a DMBA carcinogen induced tumor in chemokine receptor 3 (CXCR3) knockout C57BL/6 mouse. MOC2 is a highly aggressive metastatic tumor with negligible or no T cell infiltration in the tumors and is thus termed a “cold” tumor. MOC2 does not respond to anti-PD-1 alone or the combination of anti-PD-1 + anti-cytotoxic T lymphocyte antigen 4 (CTLA-4) antibody therapies [38], indicating it as a representative model for patients who do fail to respond to ICI immunotherapy. To determine whether TMV vaccine also effective against these murine oral cancer (MOC) models, we have prepared TMV vaccine from tumors grown in C57BL/6 mice. The MOC1 TMV vaccine was administered three days after tumor challenge as described in Figure 4A. Anti-PD-1 antibody (Clone RMP1-14) was administered starting on day 10 after tumor cell challenge. The data show that while TMV vaccine or anti-PD-1 antibody alone inhibits MOC1 tumor growth partially, the combination of both agents significantly reduced tumor growth, suggesting an additive effect (Figure 4B). The combination therapy also significantly increased survival of tumor bearing mice and eliminated tumors in 40% (2/5) of mice (Figure 4C). Tumor-free mice in the combination group were rechallenged with MOC1 tumor cells 100 days after the primary challenge to confirm durable anti-tumor immunity in vaccinated mice. We found that none of the protected mice developed tumors upon rechallenge.

**Figure 5:**
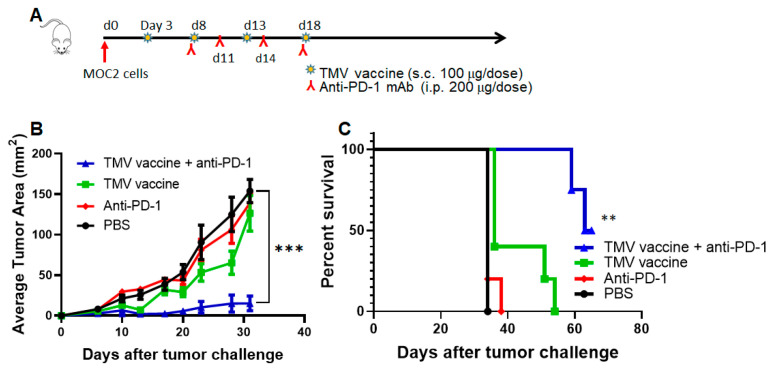
TMV vaccine + anti-PD-1 antibody inhibits MOC2 tumor growth. (**A**) C57BL/6 mice (*n* = 5) were inoculated with 5 × 10^5^ MOC2 cells s.c. on the flank. TMV vaccine was administered starting on day 3 and every five days for four doses (100 μg/dose). For the mice receiving mouse anti-mouse-PD-1 mAb, treatment started on day 8 (200 μg/dose, i.p. route) and continued every three days for four doses (inverted red Y). (**B**) Tumor size and (**C**) survival are shown. Values are Mean ± SEM and Student’s t-test between PBS and combination group. Log-rank (Mantel–Cox) test was used for determining the significance of the difference in the survival of mice. ** *p* < 0.01, *** *p* < 0.001. One outlier in the combination group is not included in the analysis.

To test whether TMV vaccine inhibits the growth of the highly aggressive MOC2 tumor growth, we administered mice with MOC2 TMV vaccine every five days starting day 3 after tumor challenge and mouse anti-mouse PD-1 antibody (kind gift from Dr. Gordon Freeman) starting on day 8 after tumor challenge, as described in Figure 5A. The data suggest that MOC2 tumor growth is not affected by anti-PD-1 antibody (Figure 5B), which is consistent with earlier studies [38] and our own studies using rat anti-mouse PD-1 antibody. Interestingly, the TMV vaccine was able to inhibit MOC2 tumor growth and synergized with anti-PD-1 antibody in controlling MOC2 tumor growth and prolonging survival of the mice, with tumors being 10 times smaller in the combination treatment group (~15 mm^2^) compared to PBS or monotherapy treated after 30 days (Figure 5C).

### 3.7. HNSCC Tumor Cells Respond to IFN-γ Treatment In Vitro

**Figure 6:**
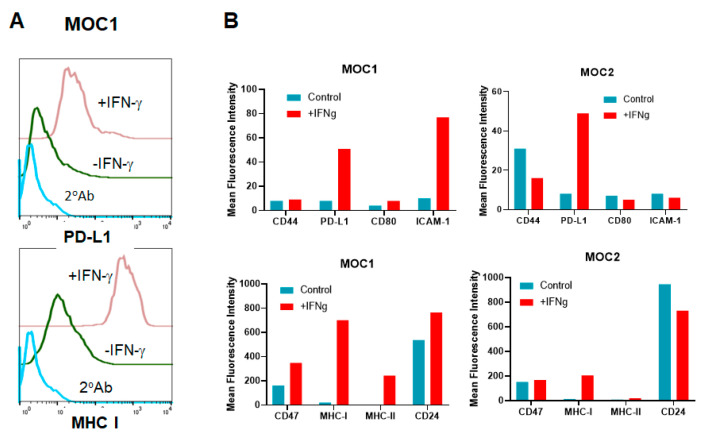
IFN-γ induces MHC class I, PD-L1, ICAM-1, and MHC class II on MOC1 but only MHC class I and PD-L1 on MOC2 cells. Tumor cells (0.5 × 10^6^ cells in 3 mL) were cultured in six-well plates with or without 100 ng/mL of IFN-γ for three days. Cells were harvested and stained for surface markers and analyzed by flow cytometry. (**A**) Representative histograms of surface PD-L1 (upper panel) and MHC class I (lower panel) on MOC1 cells. (**B**) Mean Fluorescence Intensity (MFI) of surface markers on MOC1 (left bar graphs) and MOC2 (right bar graphs) is shown. Low intensity markers on the upper panels and high intensity markers on the bottom panels are shown. The MFI is calculated by the flowcytometry as mean value from 10,000 cells. Data represent one of the two experiments.

Tumor-infiltrating immune cells secrete interferon γ (IFN-γ) in the tumor microenvironment (TME), resulting in upregulation of many immunomodulatory molecules on tumor cells. Since IFN-γ signaling mediates the tumor’s response to immunotherapy and a defect in IFN-γ signaling is one of the mechanisms of resistance to ICI therapy, it is possible that resistance of MOC2 tumor cells to anti-PD-1 antibody therapy could be attributed to a defective response to IFN-γ [39]. To determine this, we performed phenotypic analysis of MOC1 and MOC2 cells treated with IFN-γ in vitro. IFN-γ is known to induce MHC class I, MHC class II, PD-L1, ICAM-1, and PD-1 [40,41,42,43,44]. In vitro treatment of MOC1 tumor cells with IFN-γ resulted in induction of MHC class I, MHC class II, ICAM-1 and PD-L1 but had no effect on other cell surface markers such as CD44, CD47 etc. (Figure 6A,B left panels). However, IFN-γ induced the expression of only MHC class I and PD-L1, but not MHC class II and ICAM-1 on MOC2 cells (Figure 6B, right panels) [45,46,47].

## 4. Discussion

Our earlier studies using whole cell vaccines demonstrated that expression of GPI-B7-1 and GPI-IL-12 on the tumor cells induced anti-tumor protective immunity [34,48]. This approach was further improved by protein transfer of purified GPI-ISMs onto TMVs and demonstrated that a TMV vaccine consisting of GPI-ISMs induced antigen specific anti-tumor immunity [26]. Our results using syngeneic TMV vaccine induced a long-lasting anti-tumor immunity in SCC VII tumor-bearing mice (therapeutic setting), but not in naïve mice (prophylactic setting) suggesting that tumor cells themselves provide immunogenic stimulation and that response is amplified by the vaccine administration in this model. Additionally, multiple doses of vaccine reduced tumor size and prolonged survival compared to higher amounts of one or two doses of the TMV vaccine. This suggests frequent stimulation of the immune system is more effective in controlling SCC VII tumor growth. Prolonged survival of mice in the TMV vaccinated group is due to an anti-tumor memory immune response against SCC VII tumors, since re-challenge of the tumor-free mice with SCC VII cells prevented tumor growth.

PD-L1 interacts with PD-1 on T cells and inhibits anti-tumor T cell response in the TME [45,49]. Blocking the interaction using anti-PD-1 antibody prevents negative signaling and enhances the anti-tumor T cell response. A recent study has shown that anti-PD-L1 antibody therapy failed to inhibit SCC VII tumor growth, but combination treatment with MEK inhibitor and anti-PD-L1 antibody showed a synergistic effect in inhibiting tumor growth [35]. Our study demonstrates that early administration of anti-PD-1 antibody treatment inhibits SCC VII tumor growth and induces a durable protective anti-tumor immunity, suggesting that SCC VII cells are immunogenic. Lack of response to anti-PD-L1 antibody in the previous study could be due to a difference between anti-PD-1 and anti-PD-L1 antibodies. Interestingly, we observed that the timing of the anti-PD-1 mAb treatment is also critical, as early administration inhibited tumor growth (Supplementary Figure S1) whereas later administration failed to inhibit tumor growth (Figure 2). The mechanism behind this resistance of well-established tumor to anti-PD-1 antibody treatment is not clear at present. Failure of the regression of SCC VII tumors in a subset of mice (one each in TMV vaccine dose levels) that received TMV vaccine followed by anti-PD-1 mAb therapy (delayed antibody administration) suggests that SCC VII tumors induce changes in the tumor microenvironment that prevents an anti-tumor response elicitation by anti-PD-1 antibody therapy (Figure 2).

The SCC VII squamous cell carcinoma cell line is derived from the abdominal cavity of C3H mice [50,51,52], but widely used as a model for squamous cell carcinomas of head and neck cancer as it behaves similarly to human HNSCC [28,29,35,53]. In this study, we compared the efficacy of the TMV vaccine immunotherapy in SCC VII with recently available MOC models [7,31,33,37]. Although the efficacy of TMV vaccine differed in all three models of HNSCC, the TMV vaccine efficacy is more effective than anti-PD-1 antibody in the aggressive MOC2 model. While response of MOC1 cells to anti-PD-L1 mAb is not consistent in the literature [32,36,37], we did not observe any significant response to anti-PD-1 mAb under the conditions tested. Our data show a significant decrease in the tumor size in TMV vaccine plus anti-PD-1 mAb combination treatment group compared to the PBS control group. This suggests an additive effect of TMV vaccine and anti-PD-1 mAb in controlling tumor growth. TMV vaccine plus anti-PD-1 antibody therapy also extended the survival of tumor-bearing mice. Two tumor-free mice in the combination group were rechallenged with MOC1 tumor cells 100 days after the primary challenge to confirm the long-lasting anti-tumor immunity. Our data show that MOC2 tumors do not respond to mouse anti-mouse PD1 mAb, but partially respond to TMV vaccine, while combination therapy exerts profound inhibition of tumor growth. These data suggest that the TMV vaccine synergizes with anti-PD-1 mAb to further enhance the anti-tumor activity compared to TMV vaccine or anti-PD-1 alone. In vitro treatment of MOC1 and MOC2 tumor cells with IFN-γ suggest that MOC2 cells are less responsive to IFN-γ compared to MOC1, which correlates with their response to anti-PD-1 antibody in vivo. Notably, MHC class II was upregulated in MOC1 but in MOC2 cells. Further studies are needed to determine whether the differential expression of MHC class II by these cells in response to IFN-γ plays any role in response to anti-PD-1 antibody therapy.

## 5. Conclusions

In summary, our study suggests that this TMV vaccine immunotherapy platform has the potential to augment the efficacy of existing ICI. This approach can be used to develop an effective personalized vaccine immunotherapy for HNSCC by employing TMV prepared from surgically resected tumor tissue, which is easy to produce, store, and administer to patients.

## Supplementary Materials

The following are available online at https://www.mdpi.com/2076-393X/8/2/182/s1, Figure S1. Therapeutic anti-PD1 antibody inhibits SCC VII squamous cell tumor growth; Figure S2. Prophylactic TMV vaccination delays SCC VII tumor growth.
