# Supplementary material for: Tumor Membrane Vesicle Vaccine Augments the Efficacy of Anti-PD1 Antibody in Immune Checkpoint Inhibitor-Resistant Squamous Cell Carcinoma Models of Head and Neck Cancer
Source: Vaccines (Basel). 2020 Apr 14;8(2):182. doi: 10.3390/vaccines8020182 (PMC7348725; doi:10.3390/vaccines8020182)
Supplement: Supplementary file 1 [file vaccines-08-00182-s001.pdf]

vaccines

Article

Tumor Membrane Vesicle Vaccine Augments the Efficacy of Anti-PD1 Antibody in Immune Checkpoint Inhibitor-Resistant Squamous Cell Carcinoma Models of Head and Neck Cancer

Ramireddy Bommireddy [1], Luis E. Munoz [1], Anita Kumari [1], Lei Huang [1], Yijian Fan [1], Lenore Monterroza [1], Christopher D. Pack [2], Sampath Ramachandiran [2], Shaker J.C. Reddy [2], Janet Kim [1], Zhuo G. Chen [3], Nabil F. Saba [3], Dong M. Shin [3,*] and Periasamy Selvaraj [1,*]

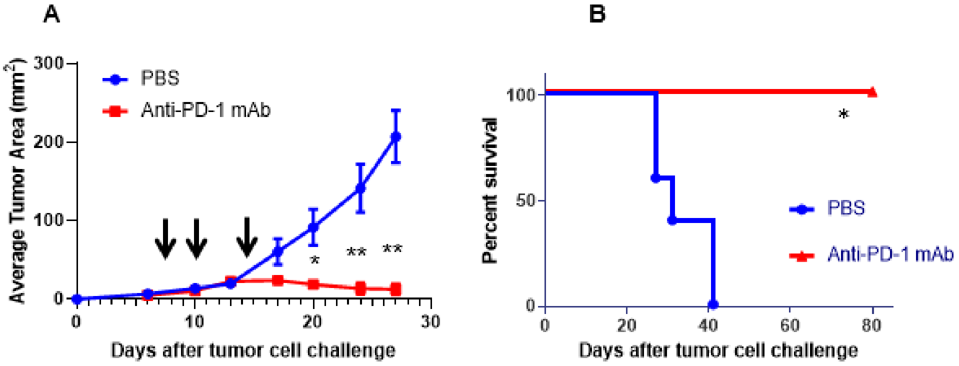

Supplementary Figure S1. Therapeutic anti-PD1 antibody inhibits SCC VII squamous cell tumor growth: (A) C3H/HeJ mice were inoculated with 4 x 10^5 SCC VII cells s.c. and were administered with 200 µg anti-PD1 antibody /mouse/dose (clone RMP1-14) starting day8 (black arrows, three doses in one week). (B) Survival of the mice was monitored. * $p < 0.02$

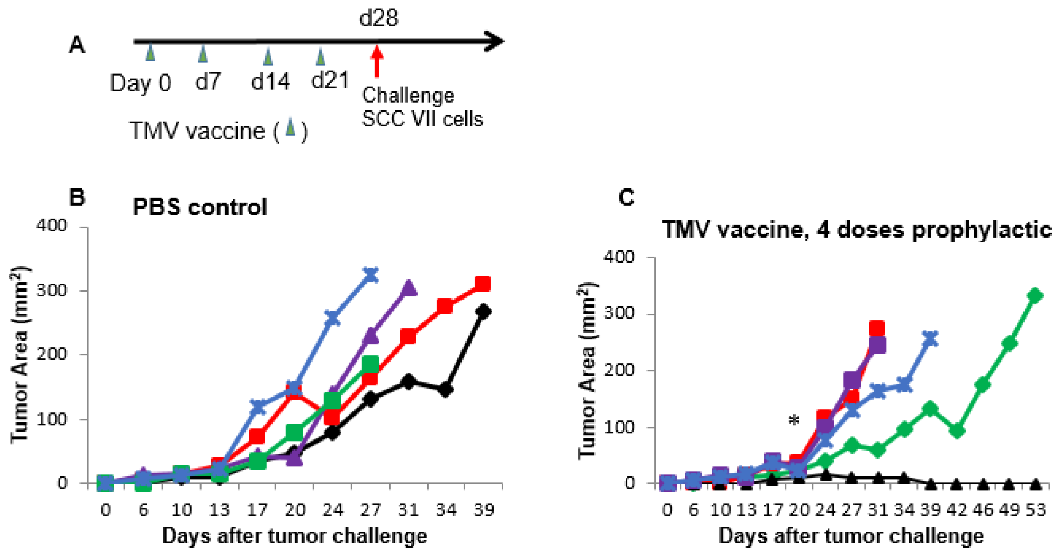

Supplementary Figure S2. Prophylactic TMV vaccination delays SCC VII tumor growth: (A) C3H/HeJ mice were administered with 100 µg TMV vaccine every week for 4 doses (d0, d7, d14 and d21) and then inoculated with 4 x 10^5 SCC VII cells s.c. on d28. Tumor size was monitored in Control PBS group (B) and TMV vaccine group (C). * $p < 0.05$

© 2020 by the authors. Submitted for possible open access publication under the terms and conditions of the Creative Commons Attribution (CC BY) license (http://creativecommons.org/licenses/by/4.0/).
